# The development of lower respiratory tract microbiome in mice

**DOI:** 10.1186/s40168-017-0277-3

**Published:** 2017-06-21

**Authors:** Nisha Singh, Asheema Vats, Aditi Sharma, Amit Arora, Ashwani Kumar

**Affiliations:** 1 Council of Scientific and Industrial Research-Institute of Microbial Technology, Sector 39 A, Chandigarh, 160036 India; 2 Council of Scientific and Industrial Research-Institute of Microbial Technology, Microbial Type Culture Collection and Gene Bank (MTCC), Chandigarh, India; 3 Present Address: Department of Medical Microbiology, PGIMER, Sector 12, Chandigarh, 160012 India

**Keywords:** Respiratory microbiome, Lung microbiome, Time series, Mice microbiome

## Abstract

**Background:**

Although culture-independent methods have paved the way for characterization of the lung microbiome, the dynamic changes in the lung microbiome from neonatal stage to adult age have not been investigated.

**Results:**

In this study, we tracked changes in composition and diversity of the lung microbiome in C57BL/6N mice, starting from 1-week-old neonates to 8-week-old mice. Towards this, the lungs were sterilely excised from mice of different ages from 1 to 8 weeks. High-throughput DNA sequencing of the 16S rRNA gene followed by composition and diversity analysis was utilized to decipher the microbiome in these samples. Microbiome analysis suggests that the changes in the lung microbiome correlated with age. The lung microbiome was primarily dominated by phyla *Proteobacteria*, *Firmicutes*, *Bacteroidetes*, and *Actinobacteria* in all the stages from week 1 to week 8 after birth. Although *Defluvibacter* was the predominant genus in 1-week-old neonatal mice, *Streptococcus* became the dominant genus at the age of 2 weeks. *Lactobacillus*, *Defluvibacter*, *Streptococcus*, and *Achromobacter* were the dominant genera in 3-week-old mice, while *Lactobacillus* and *Achromobacter* were the most abundant genera in 4-week-old mice. Interestingly, relatively greater diversity (at the genus level) during the age of 5 to 6 weeks was observed as compared to the earlier weeks. The diversity of the lung microbiome remained stable between 6 and 8 weeks of age.

**Conclusions:**

In summary, we have tracked the development of the lung microbiome in mice from an early age of 1 week to adulthood. The lung microbiome is dominated by the phyla *Proteobacteria*, *Firmicutes*, *Bacteroidetes*, and *Actinobacteria.* However, dynamic changes were observed at the genus level. Relatively higher richness in the microbial diversity was achieved by age of 6 weeks and then maintained at later ages. We believe that this study improves our understanding of the development of the mice lung microbiome and will facilitate further analyses of the role of the lung microbiome in chronic lung diseases.

**Electronic supplementary material:**

The online version of this article (doi:10.1186/s40168-017-0277-3) contains supplementary material, which is available to authorized users.

## Background

The lower respiratory tract is continuously exposed to microorganisms carried by inhaled air. In humans, the inhaled air passes through the mouth and oropharynx, carrying resident microbes from these sites to the lungs. Despite such persistent exposure to microbes, the lower respiratory tract was considered to be sterile [1–4]. This viewpoint arose from the inability to culture microbes from specimens, such as sputum or lung aspirates, using culture-based methods. Due to this prevailing view, exploration of the lung microbiome was not included in the human microbiome project [5]. However, this paradigm has been contested using culture-independent methods via next-generation sequencing of the highly conserved 16S ribosomal RNA (16S rRNA) gene marker [6]. These culture-independent methods have shown that the lower respiratory tract of healthy humans harbors a diverse microbial community [7]. The microbial community residing in the lungs undergoes significant changes in terms of composition and diversity in a number of pulmonary diseases such as cystic fibrosis [8], asthma [9, 10], and chronic obstructive pulmonary disease (COPD) [11]. It is also speculated that changes in composition and diversity of the lower respiratory tract may determine pre-disposition and severity towards lung diseases. Systematic assessment of the role of the lung microbiome in disease manifestation and its control could improve our understanding about the relevance of lung microbiome in respiratory diseases.

Current understanding of the lung microbiome has primarily arisen through analysis of microbial communities recovered in sputum samples, aspirations from intubation, tracheal aspirates, sterile brushings, and bronchoalveolar lavage samples. Although such assessments of composition and diversity of the lower respiratory tract microbiome are valuable, they are limited by potential contamination with microbial flora from the mouth and upper respiratory tract. Several studies have reported that the microbiome of the lower respiratory tract is different from that of the upper respiratory tract and oral cavity [7, 12, 13]. However, it must be noted that in humans, the main route of passage of bacteria into the lower respiratory tract are microaspirations from the oral cavity (with oral commensals) and the upper respiratory tract [14] and it remains to be understood as to why only a few selected bacteria can settle down and enrich in the lower respiratory tract. Furthermore, differences in microbiome composition were observed between bronchoalveolar lavage samples and dissected lung tissues of mice [15]. Thus, more work is required to establish the role of diversity and composition of the lower respiratory microbiome and their association with the pulmonary pathologies. Analysis of the microbiome of the lower respiratory tract and its role in pulmonary disease pathogenesis in humans is limited due to ethical concerns and the availability of limited numbers of samples. Therefore, small animal models may be utilized for establishing the lung microbiome. In these models, it is possible to physically separate the lower respiratory tract from the upper respiratory tract thereby allowing for investigation of the contribution of the lung microbiome in lung physiology. In fact, the National Institutes of Health (NIH) has recommended development of animal models to study the mechanistic aspects observed in human studies of host pulmonary interactions with the lung microbiome [16]. Such animal models have been extensively used to understand the physiological aspects associated with the gut microbiome [17]. Furthermore, evidence emerging from animal models suggests that the lung microbiome can be manipulated through inhalation of desired microbes to improve the outcome of harmful pulmonary infections [18, 19]. Although a few studies have utilized mice models to implicate the role of microbiome in lung pathologies [20, 21], one of the major unanswered questions in the field of pulmonology is whether the manipulation of the lung microbiome can be utilized to alleviate pulmonary pathologies.

Exposure to a variety of microbes during the early ages of development has been associated with higher tolerance for common allergens and a lower risk of developing immune-related diseases such as asthma at a later stage [22–25]. It can be hypothesized that the lung microbiome develops quite earlier in life and that microbiome may guide the development of the immune system. In fact, a study has conclusively demonstrated that lung microbiota can at least in part “educate” the immune system during early life [24]. Furthermore, the lung microbiome also depends on environmental factors such as geographical location, presence of animals or pets, and presence of dust [26, 27]. However, details about the compositional dynamics of the lung microbiome with age have been examined in few studies only [28–30]. As most of these studies were performed during the course of disease development, they do not provide an insight in the healthy lung microbiota. A better understanding of microbiome development inside the lung could be established using animal models, and such knowledge may further our understanding of the effects of the lung microbiome on lung diseases. In this study, we monitored the lung microbiome from postnatal to adulthood in C57BL/6N mice. We analyzed the microbiome of the lower respiratory tract from groups of neonates and tracked changes in microbial diversity into adulthood. To this end, we used deep sequencing of 16S rRNA amplified from genomic DNA isolated from the lungs of mice.

## Results

### The developing lung harbors temporally dynamic microbial diversity

**Fig. 1:**
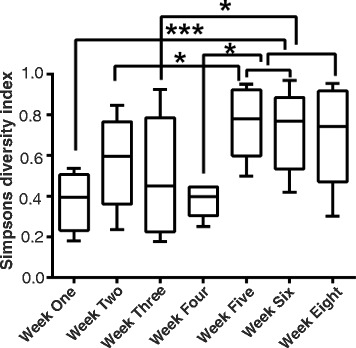
Simpson diversity for all 72 samples representing the developmental weeks. Early developmental weeks (1–4) have lower diversity than later stages (Pearson chi-squared; *p* < 0.01). The increase in diversity from week 5 can be clearly visualized from the box plots that display the following values: lower whisker, minimum; lower box border, first quartile; middle box line, median; upper box border, third quartile; and upper whisker, maximum. The *dots* represent the outlier values

The development of the lung microbiome cannot be thoroughly tracked in humans due to technical limitations and associated ethical issues. In light of these limitations, we used an inbred mouse strain for exploring the development of the lung microbiome. The use of inbred mice in this study is supported by previous studies in which mice were used as an animal model for several pulmonary diseases [31, 32]. Pulmonary development happens during early postnatal periods [33, 34], and this development may be associated with changes in the lung microbiome; therefore, we tracked the changes in the pulmonary microbiomes in mice from age of 1 week until the age of 8 weeks. It must be noted that we have not included week 7 in this work. This time point was omitted since the published literature suggest that the development of the mice body including the nervous system is finished by 6 weeks of age [35]. Furthermore, the development of the lung is also completed by 6 weeks post-birth [33, 34]. A total of 72 mice from 1 to 8 weeks old were sacrificed, and the lungs were dissected out and homogenized under sterile conditions for genomic DNA isolation. Since lung tissue represents low microbial biomass specimens and is highly vulnerable to contamination by bacterial DNA introduced at any step during harvest, processing, DNA isolation, and sequencing, we used phosphate-buffered saline in place of lung tissue as negative control. Genomic DNA isolated from the lung samples and the negative control was used to amplify the V4 variable region of the 16S rRNA gene with PCR primers targeting the +515/806 region. The primers were barcoded and PCR products were subjected to high-throughput Illumina sequencing (MiSeq). A total of 2,919,116 16S rRNA (V4 region) reads were obtained with an average of 45,037 reads across samples. Importantly, we observed only a few sequence reads in the negative control. The sequence reads were filtered and quality-checked before assigning taxonomy using Greengenes software (http://greengenes.secondgenome.com/downloads). Mapping of reads was undertaken to generate a total of 95,343 operational taxonomic units (OTUs) that could be further grouped into ~500 unique OTUs. Collectively, these sequences represented 269 unique genera. The average Shannon Diversity Index [36], taking into account both the number of OTUs and their relative abundances, for all time points ranged from 3–4, with a mean of 3.5 (confidence intervals for all the SDI values are provided in Additional file 1: Table S1). There was an overall agreement of trends when comparing between Shannon and Simpson’s diversity indices. These trends can be clearly visualized using the Simpson’s Diversity Index (SDI) [37] (Fig. 1). The Simpson diversity depicts an increase in diversity at week 2, followed by a slight decrease at weeks 3 and 4. Thereafter, diversity increases until week 5 and remains relatively constant until week 8. This trend was reproducible using the Inverse Simpson’s Diversity Index (Additional file 2: Figure S1a). The median and inter-quartile range (IQR) are provided in Additional file 2: Figure S1b.

**Fig. 2:**
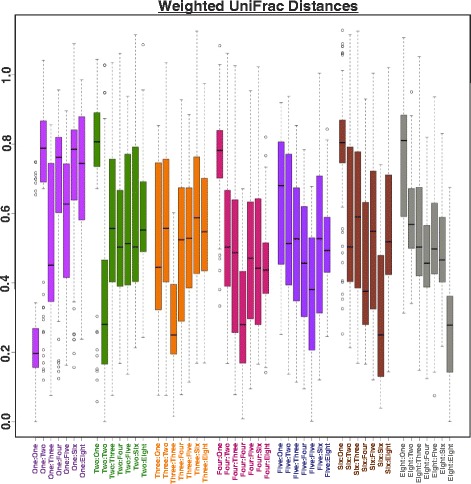
Weighted UniFrac distance box plots. The intra-week weighted UniFrac distances are smaller than the inter-week distances

To have a better understanding of the proportion of reads mapping to each genus, the abundance levels of each genus per week were represented as percentage values (Fig. 2). Hierarchical clustering of these read abundance levels (number of reads corresponds to abundance proportions—represented as percentage) revealed independent clusters of genera dominated each week, with reads mapping to weeks 4, 5, and 8 representing the largest genera clusters. Overall, the developing lung is host to diverse and dynamic microbial communities, which are dominated by clusters of unique genera (also see Fig. 7).

### Identification of the core microbial population during mice growth

**Fig. 3:**
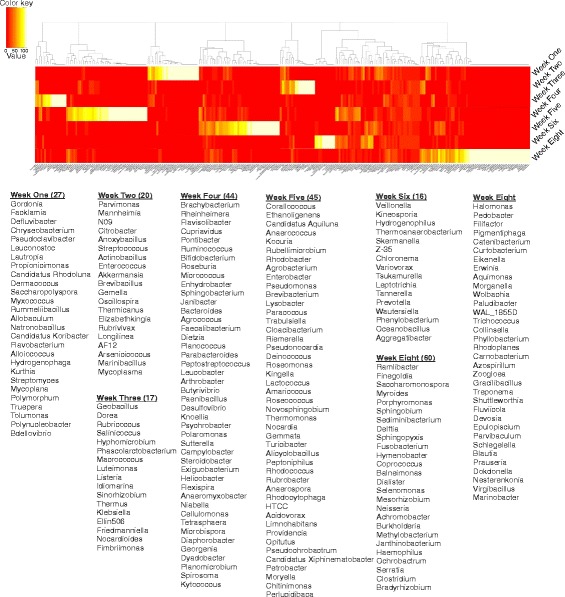
Mapping abundance levels of reads to OTUs. The proportions of reads mapping to genera, for each week, grouped into clusters that represented distinct sets of genera. Read counts were first converted to percent proportions before undertaking cluster analysis. Hierarchical clustering using Euclidean distances of read abundance levels was used to construct the heatmap. The adult mice formed the largest cluster with maximum number of reads mapping to unique OTUs. The genera with maximum abundance per week out of the 269 genera has been given below

The number of mice per week ranged from seven to 17 with week 8 (adult mice) having the maximum number of samples (Additional file 3: Table S2). We thus test the consistency of log-transformed microbial abundances (per week) in the grouping of 269 genera using variability analysis. For each of the time points studied, the intraclass correlation coefficient (ICC) iteratively accesses intra-subject similarity by comparing the variability of log-transformed abundance levels of each genus across a single week to the total variation across that week for all genera (Table 1). The consistency of ICC values (between 0.002 and 0.007) reveals that the abundance levels of the genera were evenly distributed and consistent within the groups of the animals of each week point. For further comparison of abundances across weeks, we plotted the weighted UniFrac distances for all the weeks (Fig. 3). As can be clearly seen, the intra-week weighted UniFrac distances are lesser than the inter-week weighted UniFrac distances.

**Table 1:** ICC values along with the number of mice per week

Week	ICC	No. of replicates
Week 1	0.004660935	10
Week 2	0.007651562	7
Week 3	0.002102421	10
Week 4	0.005535202	10
Week 5	0.002466764	10
Week 6	0.002825806	8
Week 8	0.005390442	17

**Fig. 4:**
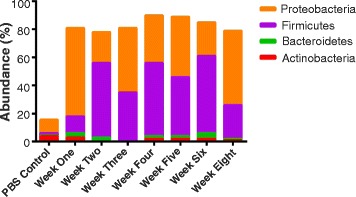
The majority of the ~500 OTUs were dominated by four phyla: *Proteobacteria*, *Firmicutes*, *Bacteroidetes*, and *Actinobacteria* were the dominant phyla in the lung microbial communities. Identities were established using sequence homology with 16SrRNA gene sequences. Different proportions of phyla can be seen at different developmental stages of the lung. More than 85% of the reads belonged to these four phyla (two-tailed *z* test; *p* < 0.01). PBS alone was used as a control which shows less abundance among the four dominant phyla

When comparing the relative percent abundance at the phyla level, more than 85% (two-tailed *z* test; *p* value <0.01) of the microbial communities in mice from week 1 to week 8 consisted of phyla *Proteobacteria*, *Firmicutes*, *Bacteroidetes*, and *Actinobacteria*. We used 97% 16S rRNA pairwise sequence identity via QIIME [38] for allocating the OTUs. During the first week, the most abundant phylum was *Proteobacteria*; however, at week 2 after birth, *Firmicutes* becomes the predominant phylum along with *Proteobacteria* (Fig. 4). Both these phyla remain predominant throughout the lung development. The mean abundance measure along with the standard error has also been plotted for individual phyla (Additional file 4: Figure S2). It is important to note that the percent abundance of different phyla varied at each week thereby indicating a dynamic microbial ecosystem in the murine lungs.

### Grouping of microbial abundance in the mice lungs shows temporal signatures

**Fig. 5:**
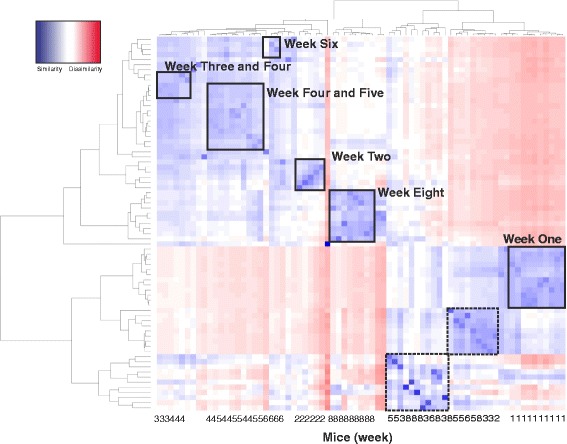
Heatmap generated and hierarchical clustering of the 72 samples: Clustering was undertaken using Ward’s method [62], and maximum linkage was applied to the log_10_-transformed abundance levels. Heatmap was generated from the weighted UniFrac distance matrix. This heatmap reveals distinct clusters for weeks 1, 2, 6, and 8. The increasing *shades of blue* denote greater similarity between samples. *Red shade* represents dissimilarity. *Squares* with *dashed border* contain single samples from different weeks. These *dashed border* clusters therefore represent the noisy grouping of samples where the neighboring samples do not belong to the same week points (represent non-specific clustering between samples). The numbers in the *X*-axis represent the week to which a particular box belong

Removal of low-abundance genera (log_10_-transformed values with abundance levels <10) from 72 samples resulted in 138 genera. This filtered dataset was used for further statistical analysis. To identify similarities and differences between the microbial communities of different samples, we initially implemented hierarchical clustering and then calculated the beta diversity indices. Cluster analyses, based on weighted UniFrac distances [39], revealed distinct clusters pertaining to weeks 1, 2, 6, and 8. Weeks 3, 4, and 5 showed an admixture affect by forming two distinct clusters (Fig. 5). Many samples clustered with other samples from the same week, thereby depicting high specificity (samples from weeks 1, 2, and 8). Samples from other weeks either clustered with nearest neighboring time point (weeks 3–4; 4–5) or non-specifically with other samples (squares with dashed border).

**Fig. 6:**
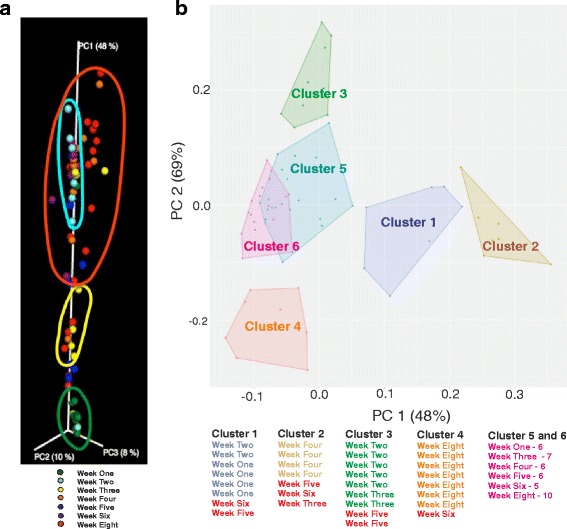
The diversity and distribution of OTUs across different developmental weeks. **a** Principal coordinate analysis (PCoA) of weighted UniFrac distances as a measure of beta diversity (between samples diversity): Samples from week 1 (*green circle*) and week 3 (*yellow circle*) grouped together into clusters (when viewed in 3D along the PC1 axis). The PCoA plot has been scaled in the direction of PC1 as it explains the maximum percent variation. **b** 2D PCA plot shows four non-overlapping clusters containing samples from two adjacent time points. The samples, shown in the *right*, are colored according to the cluster membership. Samples that do not belong to adjacent time points are colored in *red*

Weighted UniFrac distances for the principal coordinates analysis (PCoA) were used to visualize whether the samples grouped into distinct clusters as a resultant of beta diversity differences between the time points. Whereas samples from week 1 (green circle) and week 3 (yellow circle) formed distinct groups (along PC1 axis), the remaining samples (weeks 2, 4, 5, 6, and 8) (red circle) were all grouped together in a large cluster (Fig. 6a). In order to better visualize these binning, we calculated the “sum of squares” distance measures from raw abundance measures, before employing the principal component analysis (PCA) and *k*-means clustering. Four non-overlapping clusters can be seen in the 2D PCA plot (Fig. 6b). Although none of these four clusters contains unique samples from the developmental weeks, each of the clusters contains samples from two adjacent time points. This clear trend of binning alongside ones’ adjacent time point suggests the existence of a quantitative gradient in taxonomic abundance of lung metagenomic data.

**Fig. 7:**
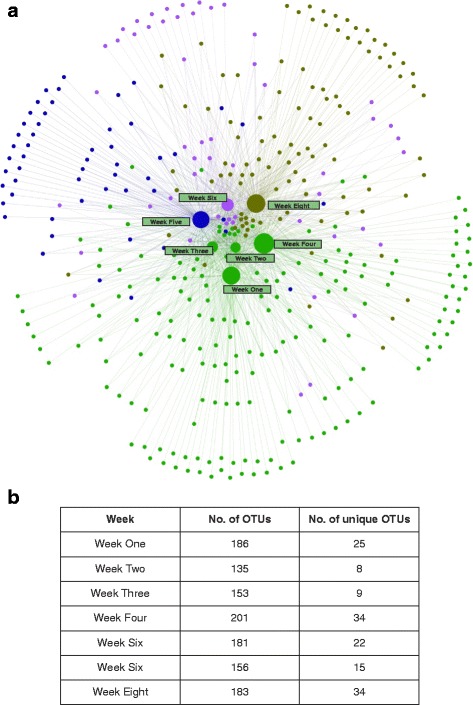
The OTU network reveals common and unique genera associated with each developmental stage. Different colors represent different modules. **a** Weeks 1, 2, 3, and 4 (*green central nodes*) formed the single largest module due to their association with shared/common OTUs, whereas weeks 4 and 8 had the most unique OTUs (*n* = 34). The OTUs (*nodes*) at the center of the network (~60) are shared by all weeks. Week nodes are scaled according to the betweenness centrality measures. **b** The table represents the total number of OTUs and the number of unique OTUs

To determine the sets of OTUs that might be common or distinct among the developmental weeks, an OTU network (no. of nodes = 399; no. of edges = 1195) linking the seven developmental weeks with the OTUs was constructed (Fig. 7a). Using force-directed graph drawing algorithm, the OTU network could be visualized for further analysis. The most “central” nodes, identified by the betweenness centrality (defined by the number of shortest paths from all nodes to all others that pass through that node), have larger nodes. These nodes represent the developmental weeks and depict the numbers of OTUs associated with each timescale. As an OTU associated with a week node could either be unique to that time point or could be shared with other week nodes, this phenomenon leads to the network having a modular structure—depicted by different colors. Modularity analysis therefore enables us to detect the communities in the network. The OTUs that are common between a single week point and the rest of the weeks, along with the unique OTUs in that particular week, form a community structure (Additional file 5: Figure S3). As seen from similar color-coding, many OTUs are shared between different week nodes, thereby imparting a modular structure to the network. Although the numbers of week points are 7, the numbers of communities are 4. Weeks 1, 2, 3, and 4 form a single large community (colored green) as the majority of OTUs they contain are shared among these 4 weeks. The colors associated with these four communities are just for visualization purposes. Analyzing nodes with single edges (leaves of the network) enables us to more clearly visualize those OTUs that are distinct for each time scale (ranges from 8 (week 2) to 34 (weeks 4 and 8), Fig. 7b). *Proteobacteria*, *Firmicutes*, *Bacteroidetes*, and *Actinobacteria* were the dominant phyla of these unique OTUs, with singular *Firmicutes* showing elevated levels at week 2 and week 3, while the unique *Proteobacteria* increased from week 5 onwards (data not shown). Betweenness centrality measure also revealed that ~60 OTUs were shared among all of the weeks (nodes at the center of the network; data not shown).

### Microbial diversity increases with the age of mice

**Fig. 8:**
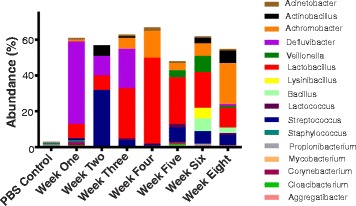
Microbial communities in the developing lung are dynamic over time. The stacked bar plot revealed that 16 genera dominated the developmental lung microbiota (test for equal proportions *p* < 0.05). The bars represent the average abundance of OTUs per week. *Lactobacillus* and *Streptococcus* were most prominent among the mice of all of the weeks. *Defluvibacter* was present in neonatal mice, while *Veillonella* started to appear at the age of 5 weeks. PBS instead of the lung tissue was used as a negative control

Employing the test for equal proportions (using Pearson’s chi-squared test statistic), a total of 16 dominant genera (*p* value <0.05) were recovered from the lungs at different weeks (Fig. 8). From this relative abundance OTU plot, it is clear that *Defluvibacter* was the predominant genus in neonatal mice at age of week 1. At this age, *Lactobacillus* was also present. At the age of week 2, *Streptococcus* becomes the dominant genus along with minor representation of *Defluvibacter* and *Lactobacillus*. At the third week after birth, *Lactobacillus*, *Defluvibacter*, and *Achromobacter* became the dominant genera and abundance of *Streptococcus* is significantly reduced. At the fourth week, *Lactobacillus* and *Achromobacter* were the most abundant genera. Interestingly, after this age, the abundance of *Defluvibacter*, which was the dominant genus at the earlier periods, is tremendously reduced. However, around an age of 5 weeks, significant increase in the microbial diversity in terms of genera was achieved. At this age, *Lactobacillus* remains the predominant genus, along with significant presence of *Streptococcus*, *Achromobacter*, *Veillonella*, *Lactococcus*, *Corynebacterium*, *Cloacibacterium*, *Acinetobacter*, and *Mycobacterium*. At an age of 6 weeks, microbial diversity further increases, with significant abundance of *Lactobacillus*, *Veillonella*, *Achromobacter*, *Streptococcus*, *Bacillus*, *Lysinibacillus*, *Actinobacillus*, *Acinetobacter*, *Propionibacterium*, and *Mycobacterium*. This richness in the microbial diversity achieved by 6 weeks is maintained to the age of 8 weeks. In all, *Lactobacillus* and *Streptococcus* were the most prominent throughout the development of the lungs. Pearson’s chi-squared test favors large differences between dominant taxa on the one hand, while exaggerating small differences between low-abundance taxa on the other. This may be considered as a disadvantage in some cases. As can be seen from Fig. 7, the 16 genera account for 50–60% of the total genera present per group. In order to clearly visualize the remaining OTUs, we are providing the bar plot for the average abundance of OTUs per week as in Additional file 6: Figure S4. The remaining large percentage is occupied by large numbers (*n* = 602) of extremely low-abundance taxa.

**Fig. 9:**
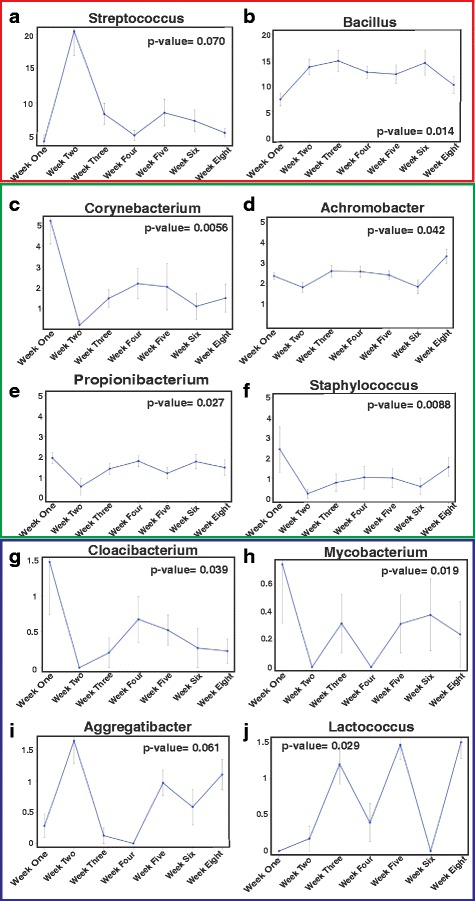
Few genera show large fluctuations in their abundance levels, across the developmental cycle. Ten genera underwent maximum temporal variation. The highly dynamic variations in these 10 genera (**a**-**j**) are depicted using average log_10_-transformed abundance levels (along with standard error values as error bars). The significant *p*-values (*p* < 0.1) of the coefficient estimates are shown on the plot corners. The genera *Mycobacterium*, *Aggregatibacter*, *Cloacibacterium*, and *Lactococcus* have low-abundance levels. For better inspection, we have divided these plots onto three sections (*red*, *green* and *blue*) reflecting their relative abundances

In order to analyze the genera with maximal temporal variation (genera that underwent large fluctuations in their abundance levels, throughout the developmental cycle), a time series was created for each of the 138 genera (week 1 to week 8). Out of the 138 genera, 40 were selected for further analysis by visually inspecting their time series and lag (=1) profiles. Considering autoregressive model of order 1 (AR1), it was feasible to employ regression analysis for further model elucidation. This resulted in the selection of 10 genera based on significant (so as to include majority of the genera with maximal temporal variation, genera with the coefficient estimates *p* < 0.1 was considered as significant) *p* values of the coefficient estimates (Fig. 9). The non-normalized absolute abundances plots were also generated (Additional file 7: Figure S5). These models depicting the change on the abundance levels over time suggest that microbial populations change significantly over time and some of these changes can be modeled using a time series analysis. Although we were able to analyze the genera that show large fluctuations in their abundance levels across the developmental time points, it must be noted that the genera *Mycobacterium*, *Aggregatibacter*, *Cloacibacterium*, and *Lactococcus* have significantly low/negligible abundance levels. These fluctuations having low-abundance levels could be attributed to sequencing and/or normalization artifacts. These temporal fluctuations of different microbial populations generally show that microbial communities in the developing lung are dynamic over time. We speculate that environmental factors such as diet, geographical locale, and gut microbiota play a role in development of the lung microbiome. Eventually, a rich diversity is achieved in mice at 5 weeks of age, and this diversity is maintained in adult mice.

### Microbial populations define and discriminate between the different stages of lung development

**Fig. 10:**
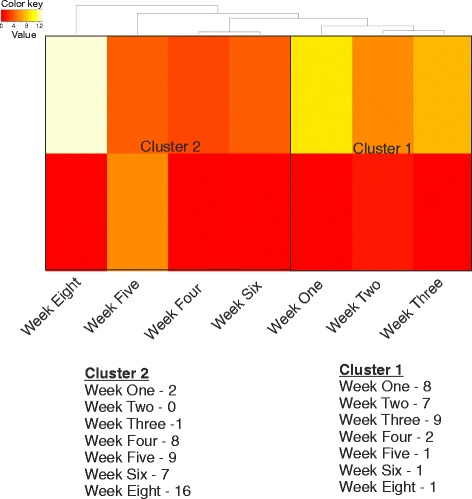
Lung microbiota discriminate between early and late developmental stages. Heatmap generated from the recursively partitioned mixture model (RPMM) for beta and Gaussian mixtures. The *columns* represent weeks, and the *rows* are the clusters. *Color* reflects the within-cluster mean abundance levels for each of the developmental stages. The different stages of lung development clustered with their respective neighbors, thereby indicating the ability of microbial populations to discriminate between the developmental weeks. Weeks 1–3 cluster together, whereas weeks 4–8 form independent clusters

As the microbial communities were dynamic over time, we further investigated whether these dynamic abundance levels could discriminate between different developmental stages. In other words, we wanted to assess the feasibility of microbial abundance levels in all samples of different developmental stages to discriminate between individual developmental stages. The recursively partitioned mixture model (RPMM) for beta and Gaussian mixtures [40] is a model-based clustering algorithm that produces a hierarchy of classes. Using a normal (Gaussian) distribution on the log_10_-transformed abundance levels, we were able to group developmental weeks according to class composition of each of the respective samples within each of the classes/clusters. Comparing the two clusters generated by RPMM, it was feasible to discriminate between different ages. As seen from Fig. 10, microbiomes from weeks 4 to 8 cluster independently. This clustering arises from richness in the diversity acquired with the age of mice. We also observed clustering of the microbiome from weeks 1 to 3, representing gradual development of diversity. Therefore, microbial communities in the lung may facilitate towards discriminating between normal developmental stages (neonatal, alveolarization, and fully developed lungs) associated with age of the mice.

## Discussion

Susceptibility towards a number of immune-related diseases such as asthma, COPD, and respiratory disease of newborns is influenced by exposure to microbes and allergens during early life. However, systematic studies relating to postnatal development of the lung with acquisition of the lung microbiome and its correlation with chronic disease susceptibility are lacking. Thus, we analyzed the composition of microbes residing in the lungs of mice at different ages to create a temporal map of microbial diversity during postnatal development. To this end, we used culture-independent high-throughput 16S rRNA pyrosequencing in order to study the developing lung microbiome. We used mice of different ages ranging from 1 to 8 weeks (neonatal to adult). We observed that the phyla *Proteobacteria*, *Firmicutes*, *Bacteroidetes*, and *Actinobacteria* dominate the lung microbiome at all the stages of development. We also demonstrated that the *Defluvibacter*, *Lactobacillus*, and *Streptococcus* are the dominant genera at the early ages (1–2 weeks), while significant richness and diversity are achieved during 4 to 6 weeks of age. The maximum diversity is achieved during the age of 5–6 weeks and then maintained in the adult life. We believe that these findings significantly enhance our understanding of lung microbiome development.

Advances in high-throughput sequencing have made composition-based microbial time series and longitudinal studies possible by analyzing temporal variations in microbial communities. These time series studies reveal unique ecological observations pertaining to microbial community stability, diversity, and dynamics. Large-scale projects such as MetaHIT primarily explore the phylogenetic composition of the healthy human microbiome while focusing on variations between individuals [41, 42]. Studies investigating temporal data are still rare, and many published studies focus on only a few time points of many subjects. Complex interactions among microbiota can either take place between microorganisms or between the microorganism and its niche environment. These factors contribute to the temporal dynamics of microbial communities. In this study, we used a variety of statistical methods to address specific aspects of the developmental lung microbiome. Using well-established statistical methodologies, such as hierarchical clustering, principal coordinates analysis (PCoA), principal component analysis (PCA), and the recursively partitioned mixture model (RPMM), we traced the dynamic changes in the lung microbiome during the development of neonatal mice into adult mice.

One of the most important findings of this study is that the microbial diversity in the mice increases with as the mice grow from a neonate into an adult. The diversity at various periods of growth was measured using well-established indices. Importantly, we observed that similar to the development of gut microbiome [43], the number of unique OTUs and their relative abundances in the lung samples increased with the age of mice. Another important finding of this study was that microbiome from mice at the similar age grouped together in the cluster analyses, based on weighted UniFrac distances. At all the ages, the lung microbiome was dominated by four phyla, namely, *Proteobacteria*, *Firmicutes*, *Bacteroidetes*, and *Actinobacteria*. Importantly, *Proteobacteria* and *Firmicutes* were the most dominant phyla at all the ages. This is noteworthy as in the healthy human lungs, *Bacteroidetes* and *Firmicutes* are the most dominant phyla [44] suggesting that the human and the mice microbiomes differ from each other in composition at the phylum level. This could be due to the fact that human subjects use nose as well as mouth for breathing, while the mice are obligate nasal breather. Moreover, at the genera level, we observed significant increase in diversity with the age. Interestingly, we observed that *Defluvibacter* and *Lactobacillus* are the predominant genera at 1 week of age. The presence of *Defluvibacter* in the lung tissue was surprising since this bacterium has not been earlier associated with the lung microbiome. On the other hand, the presence of the *Lactobacillus* has been earlier reported in human and mice. In fact, the intranasal administration of *Lactobacillus* protects from viral infections [45]. At 2 weeks of age, the microbiota of the mice lung is dominated by the *Streptococcus*. Although a number of *Streptococcus* species are known pathogens for lung infections, *Streptococcus* is one of the abundant genera that is associated with the lungs [6]. Importantly, the abundance of *Streptococcus* is tremendously reduced at the age of 3 weeks. At this age, *Defluvibacter* along with *Lactobacillus* and *Achromobacter* dominates the lung microbiota. Additionally, the presence of *Achromobacter* in the lung has been correlated with cystic fibrosis [46]. At the age of 4 weeks, a dip in the diversity was observed, whereas during the age of 5–6 weeks, higher richness was achieved at the genera levels. The diversity thus achieved was maintained at the age of 8 weeks. Importantly, we have observed the presence of number of bacteria capable of the anaerobic respiration such as *Actinobacillus*, *Veillonella*, *Lactobacillus*, *Streptococcus*, *Propionibacterium*, and *Cloacibacterium*. Anaerobic bacteria are found in the lungs, and an increase in their abundance is associated with lung pathologies such as cystic fibrosis [47]. The mechanisms and the factors that play an important role in the control of growth of these bacteria remain unknown and will play an important role in modulation of several lung pathologies. The presence of *Mycobacterium* in the lung was a surprising finding. Interestingly, atypical mycobacterium was earlier detected in the respiratory tract of adult patients with cystic fibrosis [48]. Knowing that a number of mycobacterial species are associated with lung infections, identification of the *Mycobacterium* to the species levels could be crucial for further understanding the association of *Mycobacterium* with the lungs and its effect on the development of the immune system.

Development of the lung includes morphogenesis of the alveoli and secondary septation, which is marked by an increase in the number and size of the capillaries and alveoli. This process is termed alveolarization and is considered to be a key feature of lung function. Interestingly, alveolarization occurs in postnatal stages, and development of the microbiome could be related to it. Notably, alveolarization of the lung (in mice) happens in two postnatal phases: phase 1 (day 4 to day 21 after birth), wherein new alveolar septa are formed from immature pre-existing septa, and phase 2 (14–36 days after birth), characterized by the lifting off of new alveolar septa from pre-existing septa [33, 34]. Here, we have tracked the composition of lung microbiome over various ages of mice and observed a coincidental association between the composition and diversity of the lung microbiome and the process of alveolarization. We further show that the composition of the lung microbiome is dynamic and substantial diversity is established in 4 to 6 weeks, concurrently with the completion of the second phase of alveolarization inside the lungs. Conversely, the alveolarization could be affected by the lung microbiome. An earlier study has suggested that alveolar size and number correlate with the lung microbiome [49]. Although one of the emerging hypothesis is that bacteria may influence lung growth and alveolarization, this testable hypothesis awaits further studies that could conclusively demonstrate the association of lung microbiome with postnatal development of alveolar structures. *Lactobacillus* could be used as one of the genera in experiments suggesting a role of microbiome in lung development. We believe that this issue will be addressed shortly. We also observed *Proteobacteria* and *Firmicutes* as the dominant phyla among groups of mice of different ages*.* These observations are supported by studies in adult mice where these phyla were found in the mouse lungs [15, 49]. Furthermore, it should be noted that an earlier study has observed that the murine lung microbiome influences the alveolar number and size [49]. Another study has demonstrated that lung microbiome modulates the features of asthma in mice model [20]. Importantly, another study demonstrated that vitamin D is needed for the optimal murine lung health [21]. Interestingly, it was also observed that vitamin D may influence the lung microbiota in a sex-specific fashion [21]. These observations suggest that changes in the microbiota are associated with the tolerance to allergens. Another study has demonstrated the allergen tolerance is dictated by programed death ligand-1 (PD-L1)-dependent development of Helios ^−^T regulatory cells [24]. In this study, we observed that the microbiome of 8-week-old mice was predominated by genera *Achromobacter*, *Lactobacillus*, *Streptococcus*, *Actinobacillus*, *Bacillus*, and *Veilonella*. Studies from human lung microbiome suggest that the human lung microbiome is dominated by *Streptococcus*, *Prevotella*, and *Veilonella* [50, 51]. These data appear to show that the mice lung microbiome shares partial similarity to the human lung microbiome, while harboring many other genera. This observation suggests that mouse can be used as a model for studying the lung microbiome. It will be interesting to analyze the changes in the mice lung microbiome upon induction of lung pathologies and comparing them with the available data from human clinical studies. This aspect remains beyond the scope of this study.

Although this study has tracked the changes in the composition and diversity of the lung microbiome with age, there are several important questions that remain unanswered. These include whether the lung microbiome is influenced by the sex or the weight of the mice. Some of the earlier studies have demonstrated that the gender of the mice may influence the lung microbiome [21, 52]. Since this study has not factored the effect of sex on the development of the lung, this is one of the weaknesses of the current study. Besides the weight and sex, mating experience and experience as mother also could also influence the lung microbiome of the mice. However, in this study, data about these factors was not collected and did not correlate the lung microbiome and thus represent a weakness of this study. Further studies could be performed to analyze if the abovementioned factors could influence the lung microbiome. An important observation of this study was that changes in the lung microbiome have a weak correlation with the developmental stages of alveolarization. This correlation was not further analyzed and represents another potential weakness of the current study. In future, the relationship between lung microbiome and alveolar development could be examined through inhalation of different bacterial species and analysis of the alveolar size and numbers. Additionally, inhalation of intranasal or intravenous antibiotics could be utilized to alter the lung microbiome [52] and alveolarization patterns at different ages could be studied. On the contrary, the strongest point of this study is that lung microbiome composition was correlated with the age. Towards this, the mice of different age were randomly selected and the lung microbiome analysis was performed. Several rigorous analyses were performed to conclude the relationship between the lung microbiome and the age of mice.

This study is different from other studies where the influence of the gut microbiome on lung immunity or lung diseases has been addressed [53, 54]. In this study, we have tracked the changes in the lung microbiome with the growth. However, there could be many other confounding factors that could influence the lung microbiome, including antibiotics, feed type, and stress, as seen in the gut microbiome [55]. In summary, using deep sequencing, we have tracked the changes associated with the lung microbiome at different ages from 1-week-old mice to adult age. This analysis indicates a correlation between microbial composition and alveolarization of lung in mice.

## Conclusions

Recently published literature demonstrate the presence of a unique microbiome in the lungs. Furthermore, studies have suggested that this microbiome plays an important role in protection against a number of lung pathologies. However, the development of lung microbiome with temporal resolution has been lacking. Such an understanding is essential for creating new interventions for curing the lung diseases. In the present study, we have traced the changes in the lung microbiome from neonatal (1 week) age to adulthood (8 weeks of age). The first breath of new born initiates numerous changes within the lungs of mammals and humans. We have observed that the mice lung microbiome is highly dynamic, and it undergoes major changes during the growth of the mice. During the early stages of lung development after birth, the lung microbiome is dominated by the genera, namely, *Defluvibacter*, *Streptococcus*, *Lactobacillus*, and *Achromobacter*. By 6 weeks of age, a considerable higher diversity in the composition of lung microbiome is achieved and maintained thereafter to adulthood.

## Methods

### Experimental design

The primary goal of this study is to understand the changes in composition of microbiome of the lung during the growth of mice. We studied the microbiome from week 1 onward to adulthood at 1-week intervals. We selected C57 black 6 mice, as they comprise one of the most common inbred strains of laboratory mice.

### Ethics statement

The animal experiments in this study utilized the mice and were approved by the Institutional Animal Ethics Committee of CSIR-IMTech (Approval No. IAEC/13/27). All the experiments reported herein were performed according to the guidelines issued by the Committee for the Purpose of Supervision of Experiments on Animals (No. 55/1999/CPCSEA), Ministry of Environment and Forest, Govt. of India. The mice used in this study were maintained and bred under specific pathogen-free conditions in the animal house facility of CSIR-IMTech.

### Animal breeding and selection

All the animals used in this study were housed at CSIR-IMTech animal facility. Initially, a few breeding pairs of C57BL/6N were procured from Charles River Laboratories International, USA. These pairs were used, and a colony having 200 monogamous (one male to one female) pairs was established through in-breeding. The average litter size of the animals was 5–6 mice pups. The mice were fed ad libitum feed and autoclaved water. The standard solid pellet feed (Nutrilab rodent feed from Provimi, Bangalore, India) primarily contained 3.83% moisture, 21.1% crude protein, 6.04% fat, 3.62% fiber, and 57.3% nitrogen-free extract (all *w*/*w*). The diet was verified to be pathogen-free with total bacterial count less than 100 CFU/g. The mice were housed in conventional cages. Post-birth, they were primarily fed on breast milk till the age of 2 weeks. In the third week, the animals used both solid pellet diet as well as breast milk. Animals were weaned at the age of 3–4 weeks and they used ad libitum diet. Mice were maintained at 22 ± 3 °C temperature with 12-h cycle of day and night. Similar housing conditions were provided to all the animals. For this study, at the specified age, healthy mice were randomly picked from the ~200 breeding pairs without any preference of sex, weight, or breeding experience of the breeding pair. It was ensured that the animals selected for this study at each time point were not littermate. This random selection ensured that the selected sample represented the population in general. Therefore, factors like food, water, co-house, mating experience of breeding pair, and colonization do not contribute towards the confounding effects.

### DNA extraction, amplification of 16S rRNA, and sequencing

The mice were housed at 22 ± 3 °C temperature under sterile conditions. The animals used in this study were not littermates to avoid bias arising from residing genetic inheritance. One- to 8-week-old C57Bl6 mice were sacrificed, and the lungs were isolated under aseptic conditions. The lungs were homogenized using the bead beater (Omni prep) for 30 s. Genomic DNA was isolated from the lung lysate using the Qiagen genomic DNA isolation kit. The isolated genomic DNA was sent to the MR DNA lab (Shallowater, TX, USA) for sequencing. Briefly, 515F primer (5′-GTGCCAGCMGCCGCGGTAA) and 806R primer (5′-GGACTACHVGGGTWTCTAAT-3′) were used to amplify the V4 variable region of the 16S rRNA as described earlier [56]. For amplification, a single-step 30-cycle PCR was performed using the HotStarTaq Plus Master Mix Kit (Qiagen, USA) using the below-mentioned cycling conditions: 94 °C for 3 min for denaturation, followed by 28 cycles of 94 °C for 30 s, 53 °C for 40 s, and 72 °C for 1 min, and followed by a final elongation at 72 °C for 5 min. Amplified PCR products are analyzed on 2% agarose gel. Multiple samples are pooled together, and the pooled samples were purified using calibrated Ampure XP beads. Then, the pooled and purified PCR product was used in the preparation of the DNA library by following Illumina TruSeq DNA library preparation protocol. Sequencing was performed on an Illumina MiSeq as per the manufacturer’s guidelines. Since lung samples are prone to the problems of low DNA yields in the DNA extractions, analysis of such low DNA samples is riddled with the contaminating DNA from the DNA isolation kit and sequencing. In order to eliminate bias from the contaminating DNA/low reads, we have used 10 negative controls. In these negative controls, phosphate-buffered saline was used in DNA isolation instead of the lung tissue. The resulting DNA was processed similar to the tissue samples from the lungs in 16S amplification and sequencing.

### Microbiome and statistical analysis

The primary goal of statistical analyses was to examine, in parallel, the relative abundance and diversity of the lung microbiome during developmental stages. Abundance levels, deemed to be proportional to the number of reads of a taxonomic unit per week, were generated using QIIME. Briefly, the raw reads were demultiplexed, filtered, quality-checked, and analyzed using QIIME 1.8.0 [38]. Clustering into operational taxonomic units (OTUs) was done at 97% similarity levels. The reads from the 10 negative controls were first demultiplexed using the respective barcodes and then analyzed along with the previously demultiplexed week data points. As the sequence reads from week points had been already analyzed without the 10 negative controls, the new analysis allowed us to do this analysis with the 10 negative controls. We did not find any evidence for bias or skew arising from the 10 negative control sequences. Greengenes [57] and RDP datasets [58] were employed to assign taxonomy. ICC values were calculated by using the ICCest() method of R library “ICC.” This method estimates the ICC values using the variance components from a one-way ANOVA. In order to account for uneven sample counts and low-depth samples as an artifact of sequencing, we employed standard rarefaction protocols provided in QIIME. However, it has been shown before that rarefaction may not always be the appropriate methodology to standardize all the samples [59]. Therefore, we also log_10_-transformed the sample data for statistical analysis of OTU data. Both alpha and beta diversity indices were calculated after following standard rarefactions steps for each week points. For these indices, rarefaction was done looking at the graphs of diversity vs. sequencing depth. In order to select the most appropriate sequencing depth, the first quantile value of the number of ordered reads was taken to be the threshold value. The number reads at the first quantile was 15,451 with 34,268 being the median. As the number of reads from all the 10 negative control samples were extremely low (relative to the threshold of 15451 taken for the specific week samples), this threshold value of 15451 resulted in majority of the reads being discarded. Only ~18% of the reads passed the threshold value of 15451 for PBS control. Principal coordinates analysis (PCoA) was undertaken thereafter using weighted UniFrac distances [39]. In order to better visualize PCoA’s binning, we calculated the “sum of squares” distance measures from raw abundance measures, before employing the principal component analysis and *k*-means clustering. The number of clusters was determined by employing within-group sum of squares (WSS). The cluster number (*K*) was chosen by first plotting the number of clusters vs. the WSS and then visually looking for the break point (“elbow”) in the plot. A value of *k* = 6 was chosen for further analysis. The *R* method clara (package: cluster) using “Euclidean” distances were used to define the clusters for the two-dimensional PCA plot. The OTU table generated by QIIME was further used for statistical analysis using in-house *R* (https://cran.r-project.org/) scripts. Diversity was evaluated using Simpson’s Diversity Index [60] and UniFrac distances (measure of beta diversity [61]). Both “inverse” (1/λ) and “complement” (1-λ) SDI were calculated. Higher SDI values depict greater microbial diversity. Microbial abundance levels were log_10_-transformed, and then hierarchical clustering using the “Ward.D2” [62] method and maximum linkage were used to generate the heatmap (Fig. 3). The *R* function prop.test() based on Pearson’s chi-squared test was used for equal proportions. The success and failure values were calculated from the percentage abundance values for each genus. For time series analysis, average of all the samples per week was taken. A time series was created for each of the 138 genera (week 1 to week 8). Genera were selected for further analysis by visually inspecting their time series profiles as well as lag (=1) plots. ACF (autocorrelation function) and PACF (partial correlation function) plots were generated for each of the genera to inspect whether the time series was stationary. Whenever required, differencing (for removing trend) and logs (in case of unequal variance) were taken in order to stationarize the time series. Augmented Dickey-Fuller test for stationarity was also undertaken for all the time series. As we followed the simplest autoregressive model or order 1, it was feasible to undertake linear regression analysis for the time series. The *p* value was generated from the regression model and represents the probability that the coefficient estimate is significantly different from 0.

RPMM, a model-based hierarchical clustering methodology that has been previously employed to analyze high-dimensional microbial abundance datasets [40], was used to cluster the log_10_-transformed abundances. The OTU network files generated by QIIME were input into Cytoscape (http://www.cytoscape.org/) and Gephi (https://gephi.org/). Modularity analysis, betweenness centrality, and degree indices were used to format and color the layout of the network.

## Additional files

Additional file 1: Table S1. The median and IQR values for Fig. 1. (PDF 15 kb)

Additional file 2: Figure S1. (a) Inverse SDI follows the same trend as the SDI. (b) The table represents the median and inter-quartile range (IQR). (PDF 29 kb)

Additional file 3: Table S2. The number of replicates per week and reads mapping to each sample. (PDF 180 kb)

Additional file 4: Figure S2. Represents the mean abundance measure along with the standard error for the individual phyla. (PDF 65 kb)

Additional file 5: Figure S3. The figure shows the OTUs that are common between a single week point and the rest of the weeks, along with the unique OTUs in that particular week. (PDF 470 kb)

Additional file 6: Figure S4. Represents the mean abundance measure along with the standard error for the individual genera. (PDF 61 kb)

Additional file 7: Figure S5. Represents the line plot showing the mean percent abundance measure along with the standard error for the 10 genera. (PDF 180 kb)

## Abbreviations

COPD: Chronic obstructive pulmonary disease
ICC: Intraclass correlation coefficient
OTUs: Operational taxonomic units
PCA: Principal component analysis
PCoA: Principal coordinates analysis
RPMM: Recursively partitioned mixture model
SDI: Simpson’s Diversity Index

## References

[1] Baughman RP, Thorpe JE, Staneck J, Rashkin M, Frame PT (1987). Use of the protected specimen brush in patients with endotracheal or tracheostomy tubes. Chest.

[2] Thorpe JE, Baughman RP, Frame PT, Wesseler TA, Staneck JL (1987). Bronchoalveolar lavage for diagnosing acute bacterial pneumonia. J infect Dis.

[3] Jordan GW, Wong GA, Hoeprich PD (1976). Bacteriology of the lower respiratory tract as determined by fiber-optic bronchoscopy and transtracheal aspiration. J Infect Dis.

[4] Laurenzi GA, Potter RT, Kass EH (1961). Bacteriologic flora of the lower respiratory tract. N Engl J Med.

[5] Peterson J, Garges S, Giovanni M, McInnes P, Wang L, Schloss JA, Bonazzi V, McEwen JE, Wetterstrand KA, Deal C (2009). The NIH human microbiome project. Genome Res.

[6] Beck JM, Young VB, Huffnagle GB (2012). The microbiome of the lung. Transl Res.

[7] Charlson ES, Bittinger K, Haas AR, Fitzgerald AS, Frank I, Yadav A, Bushman FD, Collman RG (2011). Topographical continuity of bacterial populations in the healthy human respiratory tract. Am J Respir Crit Care Med.

[8] Delhaes L, Monchy S, Frealle E, Hubans C, Salleron J, Leroy S, Prevotat A, Wallet F, Wallaert B, Dei-Cas E (2012). The airway microbiota in cystic fibrosis: a complex fungal and bacterial community—implications for therapeutic management. PLoS One.

[9] Huang YJ, Boushey HA (2014). The microbiome and asthma. Ann Am Thorac Soc.

[10] Hilty M, Burke C, Pedro H, Cardenas P, Bush A, Bossley C, Davies J, Ervine A, Poulter L, Pachter L (2010). Disordered microbial communities in asthmatic airways. PLoS One.

[11] Erb-Downward JR, Thompson DL, Han MK, Freeman CM, McCloskey L, Schmidt LA, Young VB, Toews GB, Curtis JL, Sundaram B (2011). Analysis of the lung microbiome in the “healthy” smoker and in COPD. PLoS One.

[12] Charlson ES, Bittinger K, Chen J, Diamond JM, Li H, Collman RG, Bushman FD (2012). Assessing bacterial populations in the lung by replicate analysis of samples from the upper and lower respiratory tracts. PLoS One.

[13] Goddard AF, Staudinger BJ, Dowd SE, Joshi-Datar A, Wolcott RD, Aitken ML, Fligner CL, Singh PK (2012). Direct sampling of cystic fibrosis lungs indicates that DNA-based analyses of upper-airway specimens can misrepresent lung microbiota. Proc Natl Acad Sci U S A.

[14] Bassis CM, Erb-Downward JR, Dickson RP, Freeman CM, Schmidt TM, Young VB, Beck JM, Curtis JL, Huffnagle GB (2015). Analysis of the upper respiratory tract microbiotas as the source of the lung and gastric microbiotas in healthy individuals. MBio.

[15] Barfod KK, Roggenbuck M, Hansen LH, Schjorring S, Larsen ST, Sorensen SJ, Krogfelt KA (2013). The murine lung microbiome in relation to the intestinal and vaginal bacterial communities. BMC Microbiol.

[16] Huang YJ, Charlson ES, Collman RG, Colombini-Hatch S, Martinez FD, Senior RM (2013). The role of the lung microbiome in health and disease. A National Heart, Lung, and Blood Institute workshop report. Am J Respir Crit Care Med.

[17] Hansen AK, Hansen CH, Krych L, Nielsen DS (2014). Impact of the gut microbiota on rodent models of human disease. World J Gastroenterol.

[18] Medina M, Vintini E, Villena J, Raya R, Alvarez S (2010). Lactococcus lactis as an adjuvant and delivery vehicle of antigens against pneumococcal respiratory infections. Bioengineered Bugs.

[19] Vintini E, Villena J, Alvarez S, Medina M (2010). Administration of a probiotic associated with nasal vaccination with inactivated *Lactococcus lactis*-PppA induces effective protection against pneumoccocal infection in young mice. Clin Exp Immunol.

[20] Remot A, Descamps D, Noordine ML, Boukadiri A, Mathieu E, Robert V, Riffault S, Lambrecht B, Langella P, Hammad H, Thomas M (2017). Bacteria isolated from lung modulate asthma susceptibility in mice. ISME J.

[21] Roggenbuck M, Anderson D, Barfod KK, Feelisch M, Geldenhuys S, Sorensen SJ, Weeden CE, Hart PH, Gorman S (2016). Vitamin D and allergic airway disease shape the murine lung microbiome in a sex-specific manner. Respir Res.

[22] Ege MJ, Mayer M, Normand AC, Genuneit J, Cookson WO, Braun-Fahrlander C, Heederik D, Piarroux R, von Mutius E (2011). Exposure to environmental microorganisms and childhood asthma. N Engl J Med.

[23] Fujimura KE, Lynch SV (2015). Microbiota in allergy and asthma and the emerging relationship with the gut microbiome. Cell Host Microbe.

[24] Gollwitzer ES, Saglani S, Trompette A, Yadava K, Sherburn R, McCoy KD, Nicod LP, Lloyd CM, Marsland BJ (2014). Lung microbiota promotes tolerance to allergens in neonates via PD-L1. Nat Med.

[25] Ownby DR, Johnson CC, Peterson EL (2002). Exposure to dogs and cats in the first year of life and risk of allergic sensitization at 6 to 7 years of age. JAMA.

[26] Stressmann FA, Rogers GB, Klem ER, Lilley AK, Donaldson SH, Daniels TW, Carroll MP, Patel N, Forbes B, Boucher RC (2011). Analysis of the bacterial communities present in lungs of patients with cystic fibrosis from American and British centers. J Clin Microbiol.

[27] Fujimura KE, Johnson CC, Ownby DR, Cox MJ, Brodie EL, Havstad SL, Zoratti EM, Woodcroft KJ, Bobbitt KR, Wegienka G (2010). Man’s best friend? The effect of pet ownership on house dust microbial communities. J Allergy Clin Immunol.

[28] Coburn B, Wang PW, Diaz Caballero J, Clark ST, Brahma V, Donaldson S, Zhang Y, Surendra A, Gong Y, Elizabeth Tullis D (2015). Lung microbiota across age and disease stage in cystic fibrosis. Sci Rep.

[29] Teo SM, Mok D, Pham K, Kusel M, Serralha M, Troy N, Holt BJ, Hales BJ, Walker ML, Hollams E (2015). The infant nasopharyngeal microbiome impacts severity of lower respiratory infection and risk of asthma development. Cell Host Microbe.

[30] Zhao J, Schloss PD, Kalikin LM, Carmody LA, Foster BK, Petrosino JF, Cavalcoli JD, VanDevanter DR, Murray S, Li JZ (2012). Decade-long bacterial community dynamics in cystic fibrosis airways. Proc Natl Acad Sci U S A.

[31] Fricker M, Deane A, Hansbro PM (2014). Animal models of chronic obstructive pulmonary disease. Expert Opin Drug Discovery.

[32] Mall MA, Graeber SY, Stahl M, Zhou-Suckow Z (2014). Early cystic fibrosis lung disease: role of airway surface dehydration and lessons from preventive rehydration therapies in mice. Int J Biochem Cell Biol.

[33] Mund SI, Stampanoni M, Schittny JC (2008). Developmental alveolarization of the mouse lung. Dev Dyn.

[34] Schittny JC, Mund SI, Stampanoni M (2008). Evidence and structural mechanism for late lung alveolarization. Am J Phys Lung Cell Mol Phys.

[35] Finlay BL, Darlington RB (1995). Linked regularities in the development and evolution of mammalian brains. Science.

[36] Shannon CE (1948). A mathematical theory of communication. Bell Syst Tech J.

[37] Simpson EH. Measurement of diversity. Nature. 1949;163:688.

[38] Caporaso JG, Kuczynski J, Stombaugh J, Bittinger K, Bushman FD, Costello EK, Fierer N, Pena AG, Goodrich JK, Gordon JI (2010). QIIME allows analysis of high-throughput community sequencing data. Nat Methods.

[39] Lozupone C, Knight R (2005). UniFrac: a new phylogenetic method for comparing microbial communities. Appl Environ Microbiol.

[40] Houseman EA, Christensen BC, Yeh RF, Marsit CJ, Karagas MR, Wrensch M, Nelson HH, Wiemels J, Zheng S, Wiencke JK, Kelsey KT (2008). Model-based clustering of DNA methylation array data: a recursive-partitioning algorithm for high-dimensional data arising as a mixture of beta distributions. BMC Bioinf.

[41] Human Microbiome Project Consortium. Structure, function and diversity of the healthy human microbiome. Nature. 2012. 486:207–14. 10.1038/nature11234 PMC3564958 22699609

[42] Qin J, Li R, Raes J, Arumugam M, Burgdorf KS, Manichanh C, Nielsen T, Pons N, Levenez F, Yamada T (2010). A human gut microbial gene catalogue established by metagenomic sequencing. Nature.

[43] Kostic AD, Gevers D, Siljander H, Vatanen T, Hyotylainen T, Hamalainen AM, Peet A, Tillmann V, Poho P, Mattila I (2015). The dynamics of the human infant gut microbiome in development and in progression toward type 1 diabetes. Cell Host Microbe.

[44] Marsland BJ, Gollwitzer ES (2014). Host-microorganism interactions in lung diseases. Nat Rev Immunol.

[45] Harata G, He F, Hiruta N, Kawase M, Kubota A, Hiramatsu M, Yausi H (2010). Intranasal administration of *Lactobacillus rhamnosus* GG protects mice from H1N1 influenza virus infection by regulating respiratory immune responses. Lett Appl Microbiol.

[46] De Baets F, Schelstraete P, Van Daele S, Haerynck F, Vaneechoutte M (2007). Achromobacter xylosoxidans in cystic fibrosis: prevalence and clinical relevance. J Cyst Fibros.

[47] Tunney MM, Field TR, Moriarty TF, Patrick S, Doering G, Muhlebach MS, Wolfgang MC, Boucher R, Gilpin DF, McDowell A, Elborn JS (2008). Detection of anaerobic bacteria in high numbers in sputum from patients with cystic fibrosis. Am J Respir Crit Care Med.

[48] Millar FA, Simmonds NJ, Hodson ME (2009). Trends in pathogens colonising the respiratory tract of adult patients with cystic fibrosis, 1985–2005. J Cyst Fibros.

[49] Yun Y, Srinivas G, Kuenzel S, Linnenbrink M, Alnahas S, Bruce KD, Steinhoff U, Baines JF, Schaible UE (2014). Environmentally determined differences in the murine lung microbiota and their relation to alveolar architecture. PLoS One.

[50] Molyneaux PL, Cox MJ, Willis-Owen SA, Mallia P, Russell KE, Russell AM, Murphy E, Johnston SL, Schwartz DA, Wells AU (2014). The role of bacteria in the pathogenesis and progression of idiopathic pulmonary fibrosis. Am J Respir Crit Care Med.

[51] Dickson RP, Erb-Downward JR, Huffnagle GB (2013). The role of the bacterial microbiome in lung disease. Expert Rev Respir Med.

[52] Barfod KK, Vrankx K, Mirsepasi-Lauridsen HC, Hansen JS, Hougaard KS, Larsen ST, Ouwenhand AC, Krogfelt KA (2015). The murine lung microbiome changes during lung inflammation and intranasal vancomycin treatment. Open Microbiol J.

[53] Russell SL, Gold MJ, Hartmann M, Willing BP, Thorson L, Wlodarska M, Gill N, Blanchet MR, Mohn WW, McNagny KM, Finlay BB (2012). Early life antibiotic-driven changes in microbiota enhance susceptibility to allergic asthma. EMBO Rep.

[54] Russell SL, Gold MJ, Reynolds LA, Willing BP, Dimitriu P, Thorson L, Redpath SA, Perona-Wright G, Blanchet MR, Mohn WW (2015). Perinatal antibiotic-induced shifts in gut microbiota have differential effects on inflammatory lung diseases. J Allergy Clin Immunol.

[55] Hufeldt MR, Nielsen DS, Vogensen FK, Midtvedt T, Hansen AK (2010). Variation in the gut microbiota of laboratory mice is related to both genetic and environmental factors. Comp Med.

[56] Caporaso JG, Lauber CL, Walters WA, Berg-Lyons D, Lozupone CA, Turnbaugh PJ, Fierer N, Knight R (2011). Global patterns of 16S rRNA diversity at a depth of millions of sequences per sample. Proc Natl Acad Sci U S A.

[57] Wang Q, Garrity GM, Tiedje JM, Cole JR (2007). Naive Bayesian classifier for rapid assignment of rRNA sequences into the new bacterial taxonomy. Appl Environ Microbiol.

[58] Cole JR, Chai B, Farris RJ, Wang Q, Kulam-Syed-Mohideen AS, McGarrell DM, Bandela AM, Cardenas E, Garrity GM, Tiedje JM (2007). The ribosomal database project (RDP-II): introducing myRDP space and quality controlled public data. Nucleic Acids Res.

[59] McMurdie PJ, Holmes S (2014). Waste not, want not: why rarefying microbiome data is inadmissible. PLoS Comput Biol.

[60] Hill MO (1973). Diversity and evenness: a unifying notation and its consequences. Ecology.

[61] Lozupone CA, Knight R (2008). Species divergence and the measurement of microbial diversity. FEMS Microbiol Rev.

[62] Murtagh F, Legendre P (2014). Ward’s hierarchical agglomerative clustering method: which algorithms implement ward’s criterion?. J Classif.

