# Erratum to: The development of lower respiratory tract microbiome in mice

**DOI:** 10.1186/s40168-017-0335-x

**Published:** 2017-09-21

**Authors:** Nisha Singh, Asheema Vats, Aditi Sharma, Amit Arora, Ashwani Kumar

**Affiliations:** 1 Council of Scientific and Industrial Research-Institute of Microbial Technology, Sector 39 A, Chandigarh, 160036 India; 2 Council of Scientific and Industrial Research-Institute of Microbial Technology, Microbial Type Culture Collection and Gene Bank (MTCC), Chandigarh, India; 3 Present Address: Department of Medical Microbiology, PGIMER, Sector 12, Chandigarh, 160012 India

## Erratum

Since publication of their article [1], the authors reported that a requested proof correction had not been carried out in the final published version. The authors had asked for Figure Numbers 2 and 3 to be swapped, and the published version should reflect this.

The original article has been corrected.
