# Supplementary material for: The development of lower respiratory tract microbiome in mice
Source: Microbiome. 2017 Jun 21;5:61. doi: 10.1186/s40168-017-0277-3 (PMC5479047; doi:10.1186/s40168-017-0277-3)
Supplement: Supplementary file 1 — The median and IQR values for Fig. 1. (PDF 15 kb) [file 40168_2017_277_MOESM1_ESM.pdf]

**Week**	**Median**	**IQR**
Week One	0.3394	0.19747
Week Two	0.5785	0.31513
Week Three	0.44997	0.3378
Week Four	0.42004	0.2964
Week Five	0.752256	0.19884
Week Six	0.761914	0.1861
Week Eight	0.742544	0.2411

Table S1
