# Supplementary figures and images for: The development of lower respiratory tract microbiome in mice
Source: Microbiome. 2017 Jun 21;5:61. doi: 10.1186/s40168-017-0277-3 (PMC5479047; doi:10.1186/s40168-017-0277-3)

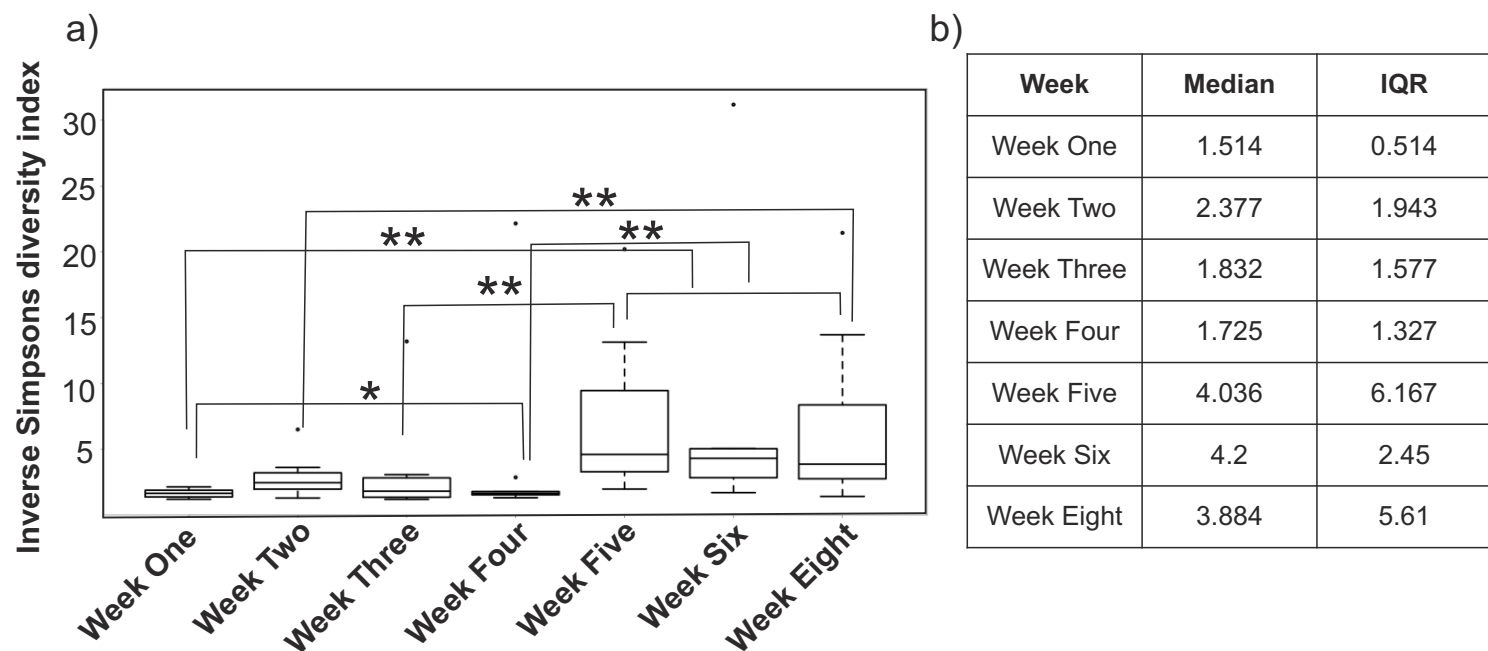

Figure S1

Supplement: Supplementary file 2 — (a) Inverse SDI follows the same trend as the SDI. (b) The table represents the median and inter-quartile range (IQR). (PDF 29 kb) [file 40168_2017_277_MOESM2_ESM.pdf]

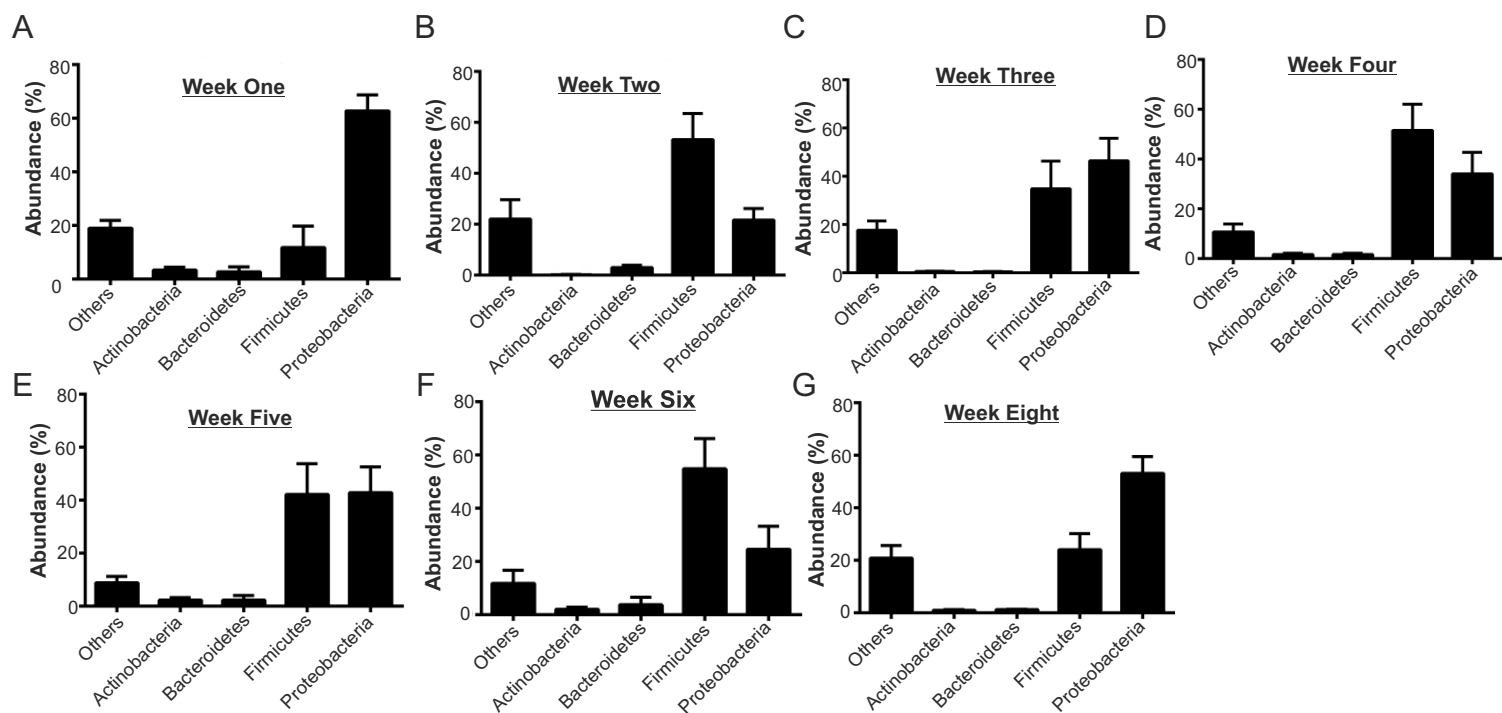

Figure S2

Supplement: Supplementary file 4 — Represents the mean abundance measure along with the standard error for the individual phyla. (PDF 65 kb) [file 40168_2017_277_MOESM4_ESM.pdf]

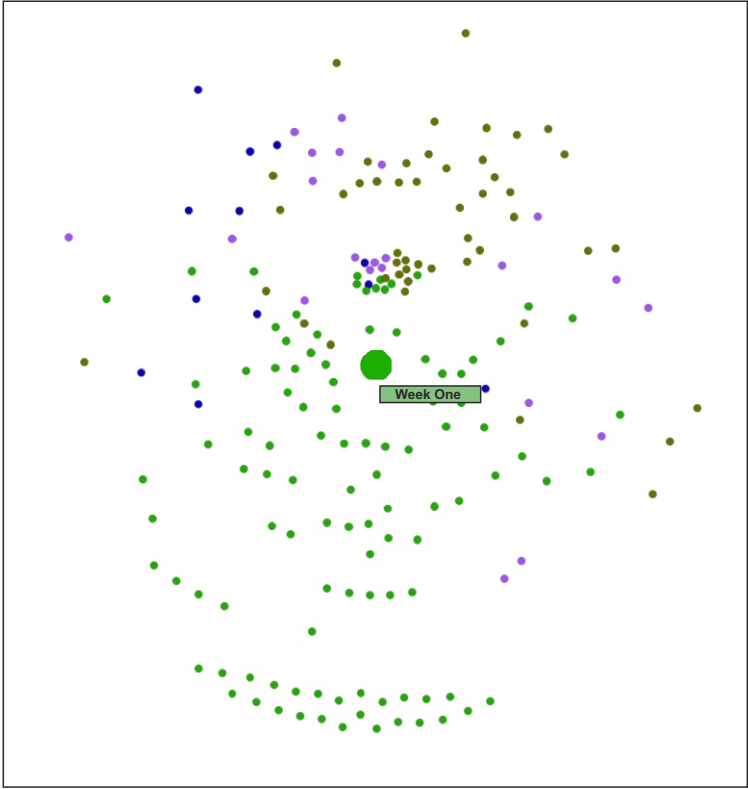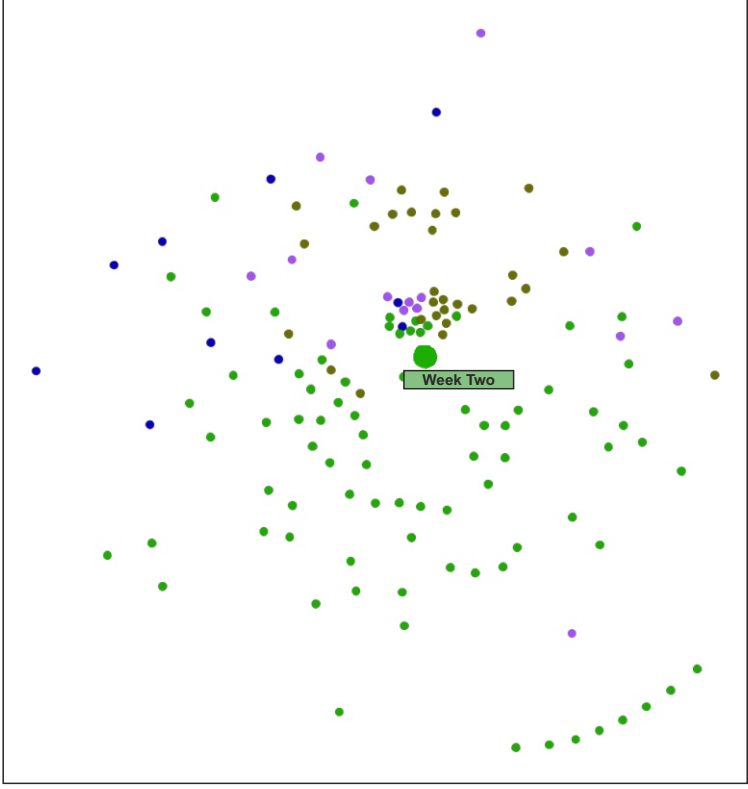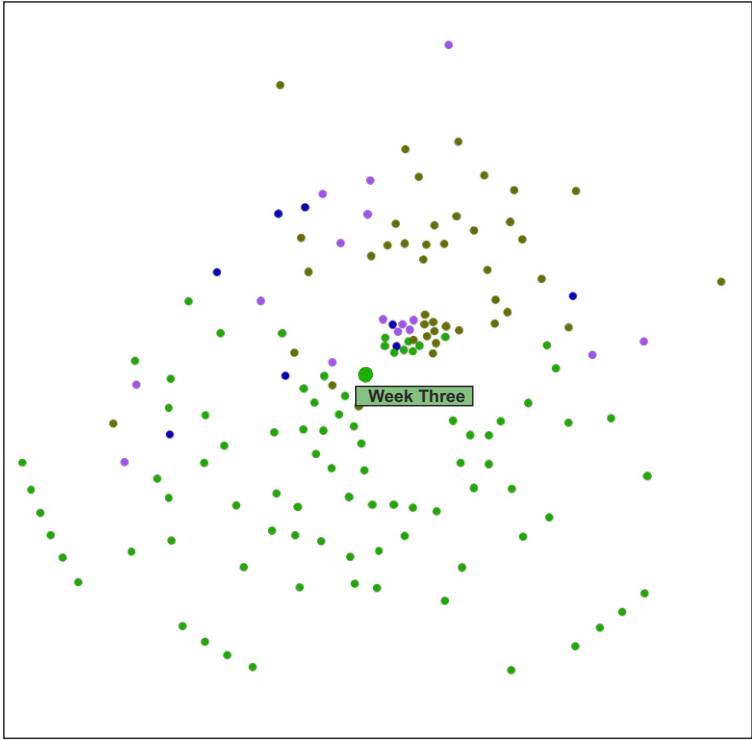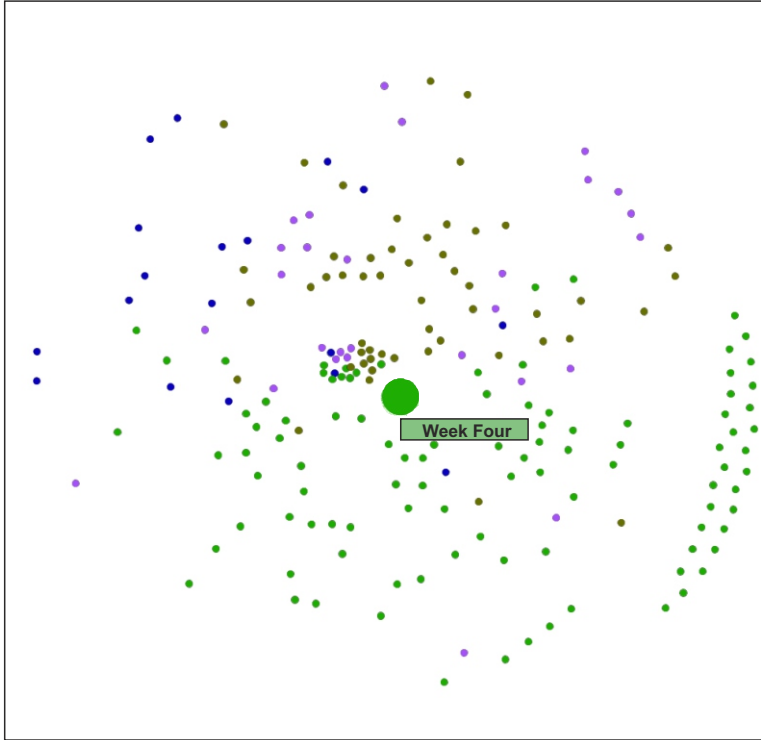

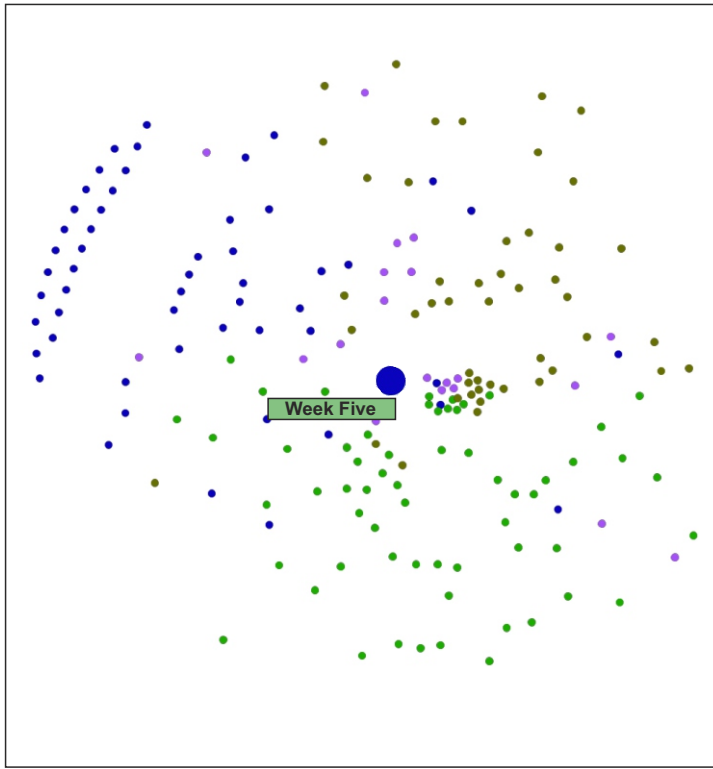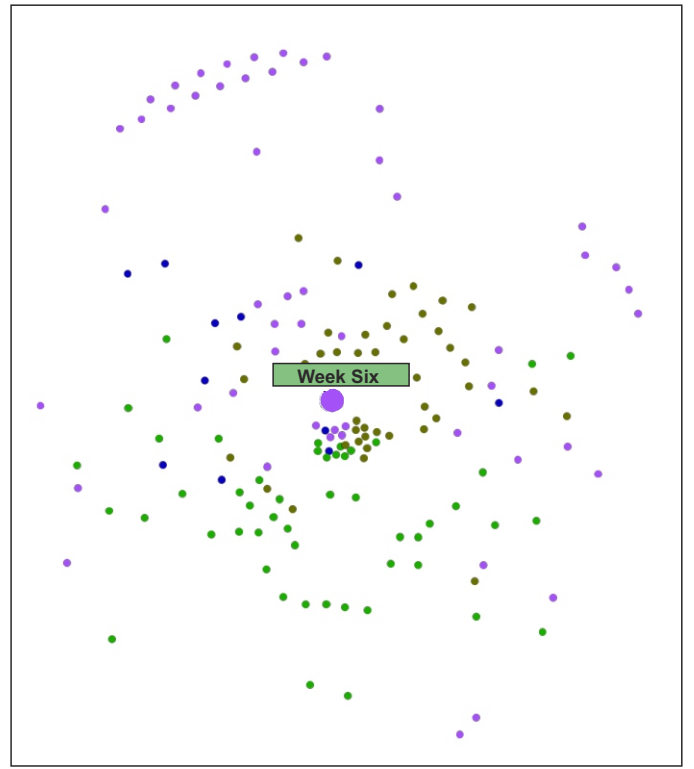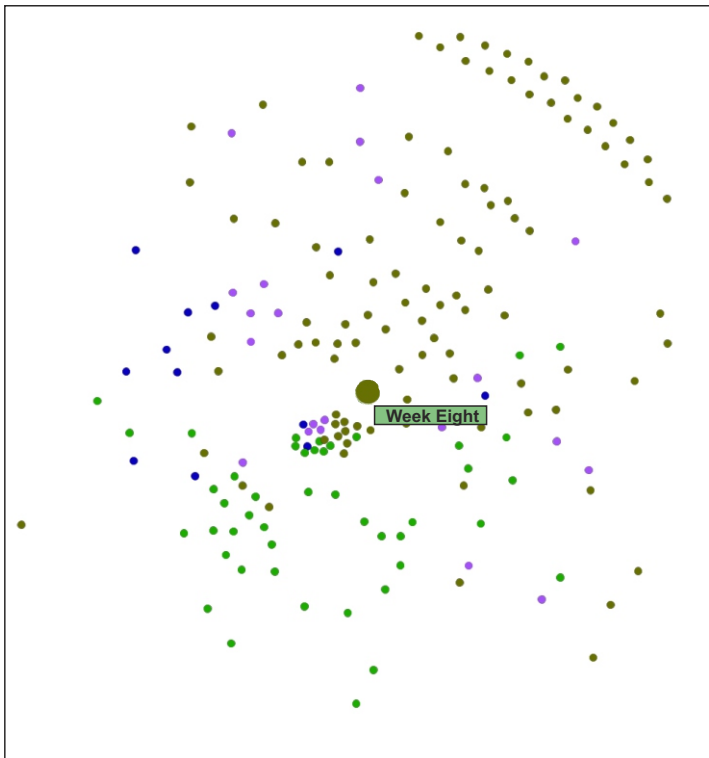

Figure S3

Supplement: Supplementary file 5 — The figure shows the OTUs that are common between a single week point and the rest of the weeks, along with the unique OTUs in that particular week. (PDF 470 kb) [file 40168_2017_277_MOESM5_ESM.pdf]

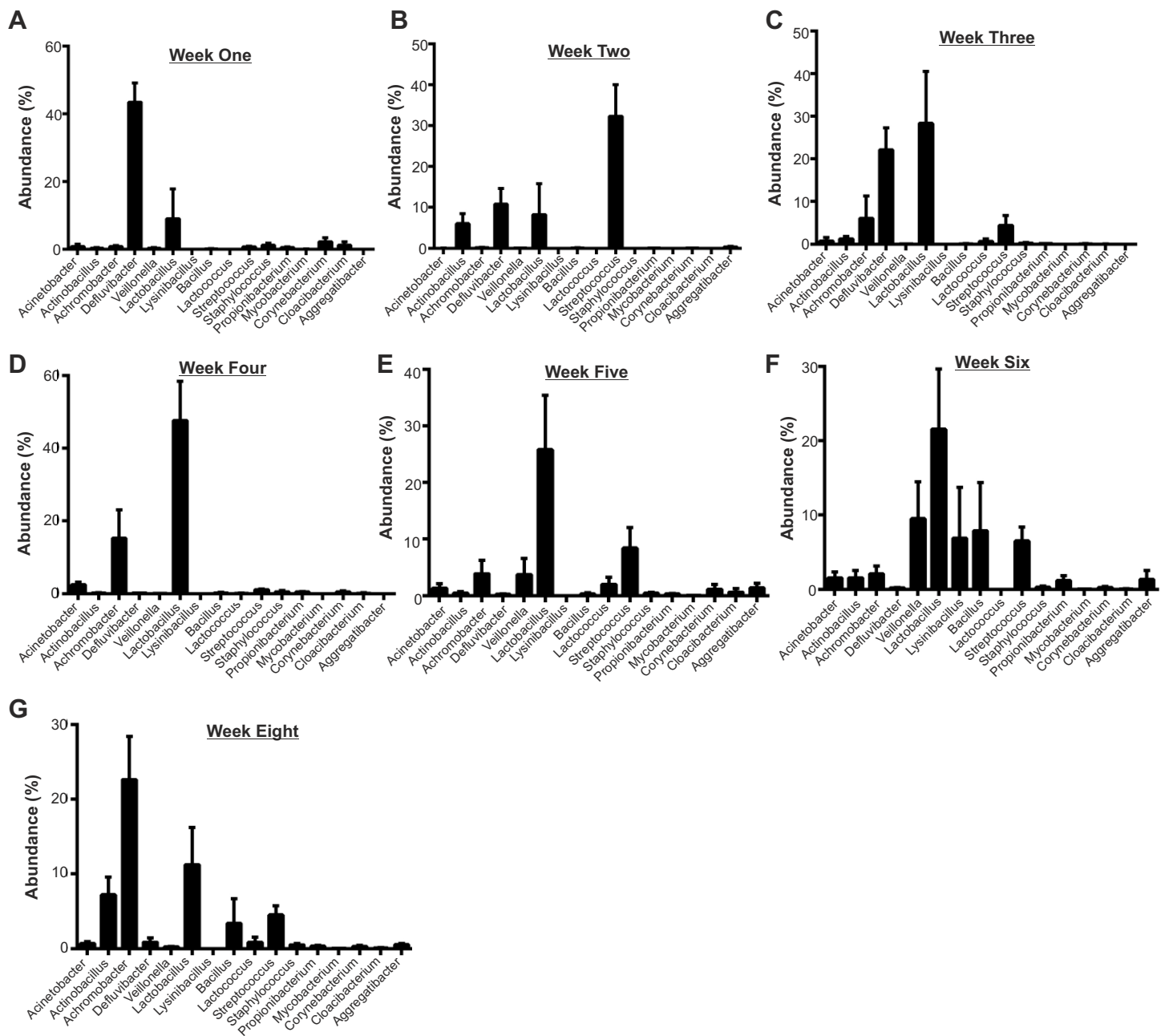

Figure S4

Supplement: Supplementary file 6 — Represents the mean abundance measure along with the standard error for the individual genera. (PDF 61 kb) [file 40168_2017_277_MOESM6_ESM.pdf]

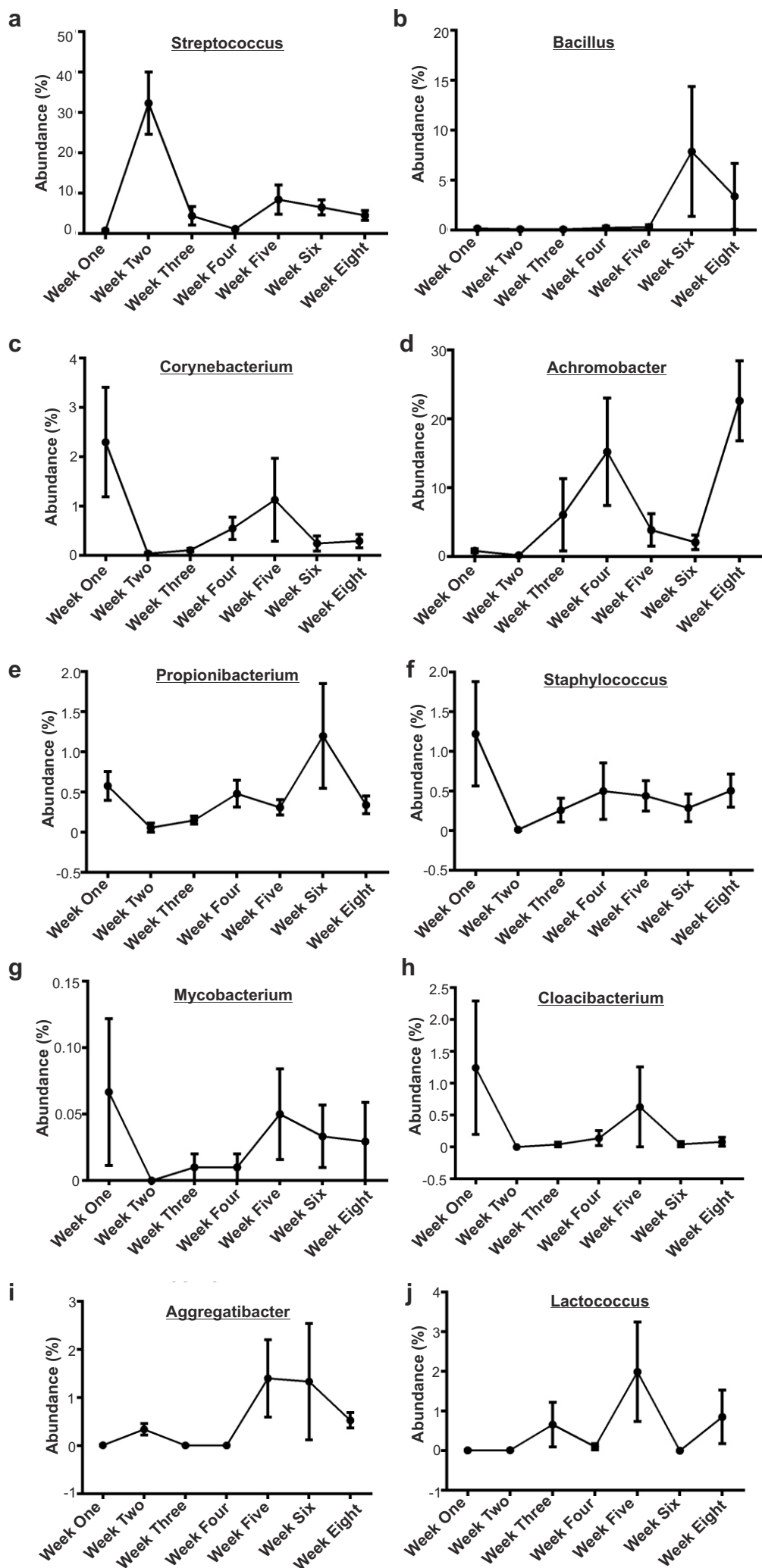

Figure S5

Supplement: Supplementary file 7 — Represents the line plot showing the mean percent abundance measure along with the standard error for the 10 genera. (PDF 180 kb) [file 40168_2017_277_MOESM7_ESM.pdf]
