# Supplementary material for: The development of lower respiratory tract microbiome in mice
Source: Microbiome. 2017 Jun 21;5:61. doi: 10.1186/s40168-017-0277-3 (PMC5479047; doi:10.1186/s40168-017-0277-3)
Supplement: Supplementary file 3 — The number of replicates per week and reads mapping to each sample. (PDF 180 kb) [file 40168_2017_277_MOESM3_ESM.pdf]

S.No	Week One	Week Two	Week Three	Week Four	Week Five	Week Six	Week Eight
1	7057	135756	7751	27898	3242	8441	997
2	13569	44405	10084	29545	3795	9663	2545
3	15881	34296	26460	31308	5837	26469	4549
4	18966	46833	31945	32796	20279	34268	6375
5	29314	91101	36715	39799	39748	55022	7282
6	36961	91603	38579	52737	51198	94614	7306
7	37111	130864	46214	57087	39267	121184	12574
8	39744		59704	60625	53700	122587	15451
9	45135		80448	62153	55933		18126
10	98045		95624	13386	98274		18454
11							20313
12							20585
13							21480
14							32893
15							39641
16							43363
17							46141
Count	10	7	10	10	10	8	17
Average	34178	82123	43352	40733	37128	59031	18710
Sum	341783	574858	433524	407334	37127	472248	318092
Total reads	2919116						

Table S2
